# A Framework for Analyzing and Measuring Usage and Engagement Data (AMUsED) in Digital Interventions: Viewpoint

**DOI:** 10.2196/10966

**Published:** 2019-02-15

**Authors:** Sascha Miller, Ben Ainsworth, Lucy Yardley, Alex Milton, Mark Weal, Peter Smith, Leanne Morrison

**Affiliations:** 1 Center for Clinical and Community Applications of Health Psychology Department of Psychology University of Southampton Southampton United Kingdom; 2 Department of Psychology University of Bath Bath United Kingdom; 3 School of Psychological Science University of Bristol Bristol United Kingdom; 4 Web and Internet Science Group School of Electronics and Computer Science University of Southampton Southampton United Kingdom; 5 Department of Social Statistics and Demography School of Economic, Social and Political Sciences University of Southampton Southampton United Kingdom; 6 Primary Care and Population Sciences School of Medicine University of Southampton Southampton United Kingdom

**Keywords:** behavioral research, internet, health, patient engagement, data analysis

## Abstract

Trials of digital interventions can yield extensive, in-depth usage data, yet usage analyses tend to focus on broad descriptive summaries of how an intervention has been used by the whole sample. This paper proposes a novel framework to guide systematic, fine-grained usage analyses that better enables understanding of how an intervention works, when, and for whom. The framework comprises three stages to assist in the following: (1) familiarization with the intervention and its relationship to the captured data, (2) identification of meaningful measures of usage and specifying research questions to guide systematic analyses of usage data, and (3) preparation of datasheets and consideration of available analytical methods with which to examine the data. The framework can be applied to inform data capture during the development of a digital intervention and/or in the analysis of data after the completion of an evaluation trial. We will demonstrate how the framework shaped preparation and aided efficient data capture for a digital intervention to lower transmission of cold and flu viruses in the home, as well as how it informed a systematic, in-depth analysis of usage data collected from a separate digital intervention designed to promote self-management of colds and flu. The Analyzing and Measuring Usage and Engagement Data (AMUsED) framework guides systematic and efficient in-depth usage analyses that will support standardized reporting with transparent and replicable findings. These detailed findings may also enable examination of what constitutes effective engagement with particular interventions.

## Introduction

Digital interventions are intended to support positive change in a range of health-related outcomes, including psychological, behavioral, educational, social, and environmental [1-3]. They may be delivered using any digital device (eg, phone and computer), making them cost-effective for providers [4,5]. Trialing a digital intervention can yield complex, large-scale datasets containing detailed usage data. If analyzed appropriately, this data is able to provide invaluable detail on how users interact with the intervention and inform our understanding of engagement. Measuring digital intervention engagement has been described as a multidimensional concept, including the extent to which an intervention is used (eg, amount, frequency, and duration) and the subjective experience of the user as characterized by attention, affect, and interest [6]. As a key element of engagement, in-depth and consistently applied usage analyses are capable of providing invaluable insight into the field of engagement with digital interventions.

Usage analyses frequently examine the extent to which an intervention is used by the whole sample, utilizing variables such as the number of times users logged in, total time spent on an intervention, or number of pages viewed [7]. These broad-level analyses do not always take advantage of the detailed and comprehensive data available and they frequently assume that greater amounts of usage are indicative of higher levels of interaction that lead to increased changes in target behavior [8]. Harnessing the full range of data can instead enable more informative usage variables to be computed or combined, which may answer specific research questions about patterns of usage (ie, who the intervention was used by and how it was used) [8,9]. Recent interest in *effective engagement* considers these individual patterns of usage and the minimum level of engagement necessary for changes in target behavior to occur, including variation across individuals [8,10-12]. Effective engagement is defined as sufficient engagement with the intervention to achieve intended outcomes [8]. For example, a digital intervention designed to lower the transmission of cold and flu provided four sessions of content, requiring the intervention to be accessed on four separate occasions. However, analysis of usage data, reported behavior, and incidences of illnesses revealed that using the first session alone facilitated the required change in behavior to increase positive outcomes [11]. Alternatively, effective engagement may be context dependent, whereby viewing specific content components, in a certain order, or at an appropriate time, is the minimum threshold necessary for change [8,11,12].

During the planning of a digital intervention, processes such as logic models and guiding principles may be used to structure the theoretical underpinning and associated content for the intervention [13-15]. These techniques help identify behavioral determinants (eg, beliefs associated with the target behavior), which may be important in influencing the target behavior; for example, low confidence to manage symptoms or perceived barriers to performing a specific behavior. In order to influence favorably these behavioral determinants, content containing behavior change techniques (BCTs), such as goal setting, feedback on behavior, or habit formation, are incorporated into the intervention [16]. However, by performing analyses which focus solely on broad usage patterns across the whole sample, the opportunity is missed to understand how specific intervention content (eg, BCTs) is used by subgroups with particular characteristics (ie, behavioral determinants) and the extent to which there is a relationship to the target behavior [11,16]. Devising a plan of analysis to answer these questions using the fine-grained data often available from digital interventions enables us to examine the constructs of the logic model and further our understanding of the mechanisms of action underlying successful behavior change [17,18].

Analyzing usage metrics to better understand engagement has been proposed for some time, with the “law of attrition” being one of the first theories to draw attention to the benefits of examining usage data in this way [19]. The importance of the type of content viewed as well as the amount has also been acknowledged [20]. More recently, researchers have advocated using complex log data from digital interventions to further our understanding of engagement [11,12] and to examine relationships between usage, participant characteristics, and health outcomes [21]. However, the importance of providing consistently reported findings that will enable comparison of usage across different digital interventions has also been highlighted [6,8]. Existing guidelines encourage precise and standardized reporting for general analyses of digital interventions [22,23]. The challenge of undertaking efficient and systematic analysis of large datasets without the guidance of a framework is already acknowledged: Sieverink et al detailed the importance of using research questions to guide analysis of log data [24] and Taki et al demonstrated how categorizing different usage metrics can inform our understanding of engagement [25]. However, systematic reviews suggest that these types of analysis of usage data are not yet routinely undertaken [7,26]. This may be due to the absence of a framework that contains comprehensive checklists combining both the systematic breakdown of usage data and the formulation of research questions to structure usage analyses of digital interventions. In addition, without prior identification of necessary data capture processes, the final usage data collected may be unable to answer the research questions posed.

This paper proposes a novel framework to structure the process of analyzing usage associated with a digital intervention by doing the following: (1) drawing together potential measures of usage and identifying which are meaningful to the intervention, (2) generating specific research questions to act as testable hypotheses, and (3) supporting data preparation and selection of methods for analysis. Specifically, the framework for Analyzing and Measuring Usage and Engagement Data (AMUsED) can encourage the collection and/or extraction of data that will explain who used which parts of the intervention at what time and whether that was associated with positive outcomes. The framework focuses on usage as a key component of engagement, but does not aim to encompass all aspects of engagement. Nonetheless, the examination and analyses of usage data, using the framework, can move toward the identification of what constitutes effective engagement. In addition, the framework offers an approach to digital intervention data analysis that can be applied both before and after data collection.

When used during intervention development, the AMUsED framework aids development teams to compile an a priori analysis plan for use after data collection. This allows the opportunity to evaluate whether all necessary data will be collected and whether this is in a suitable format for analysis at a later date. This is particularly pertinent for interventions that are developed with external partners who may be unaware of the theoretically based elements of the intervention and their implications for analyses. When applied after data collection, the framework is especially useful for general orientation when a researcher is unfamiliar with the intervention or when no advance plan of analysis is available. Using the framework helps focus exploratory usage analyses on addressing the theory underpinning an intervention and the plausible mechanisms of action on target outcomes, aiding more scientifically rigorous analyses. Should an analysis plan be available, the framework facilitates a review to ensure that the plan is still appropriate and aids revision where necessary.

## Development of the Framework

The AMUsED framework was initially developed as a means to systematically and rigorously analyze post hoc usage data collected during digital intervention trials. The first author (SM) was tasked with analyzing usage data from *Internet Dr*, a successfully trialed digital intervention (see the case study involving Internet Dr below). This task was challenging because of the author’s unfamiliarity with the intervention, the depth and complexity of data collected, and the absence of an existing framework to provide step-by-step guidance on approaching a usage analysis. Stages 1 and 2 of the framework were developed alongside the process of understanding and beginning analyses of usage data collected from Internet Dr. An early version of the framework was presented to a multidisciplinary digital intervention development team with experience across health psychology, primary care, and statistics. The framework was then refined based on the team’s input and experiences of applying the framework to their own usage analyses. The value of having a systematic process through which to consider data collection during the development phase of a digital intervention was subsequently noted. The framework was then expanded and applied to the amendment of a second intervention, *Germ Defence* (see the case study involving Germ Defence below). Following this, the framework was presented to the wider scientific community at a national conference in the United Kingdom. Here, the value of using the structure provided by the framework to support collaboration between social scientists and software development companies and identification of necessary data collection processes was recognized. The framework was then shaped further to provide equal weight to both a priori and post hoc analysis needs.

## Description of the Analyzing and Measuring Usage and Engagement Data (AMUsED) Framework

### Overview

**Figure 1:**
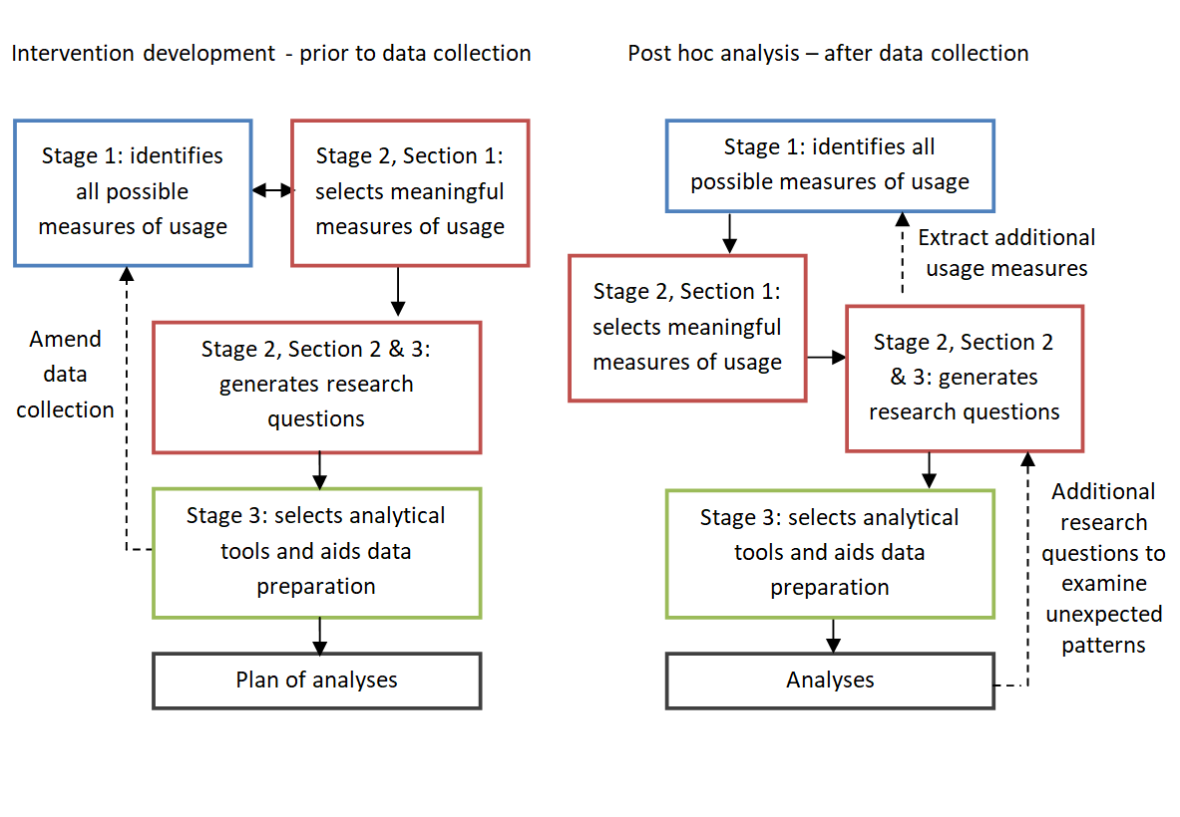
Stages of the Analyzing and Measuring Usage and Engagement Data (AMUsED) framework. Dotted lines indicate optional paths to revisit if necessary.

The framework is presented in three stages: (1) familiarization with available datasets, (2) selecting meaningful measures of usage and generating research questions, and (3) preparation for analysis. Each stage is available in checklist format, with generic questions acting as prompts for the researcher to consider in the context of their own specific intervention (see Multimedia Appendices 1-3). It is anticipated that use of the three stages will be iterative depending upon whether the framework is being applied in advance of or after data collection (see Figure 1). For example, when considering appropriate analytical software (Stage 3) during the development phase of an intervention, it may be necessary to reformat how data are recorded to ensure compatibility. Alternatively, analyses of collected data may reveal unexpected patterns of usage, such as repeated visits to a component of content, from which new exploratory research questions can then be generated (Stage 2).

The framework focuses specifically on examining the relationships and associations between measures of usage and user characteristics, theoretical variables, behavior, and/or health-related outcomes. However, it is anticipated that analyses of usage would be considered in the context of a broader process evaluation that may examine how variables other than usage are associated with intervention outcomes [27].

### Stage 1: Familiarization With the Data—Identifying Variables

Evaluation of a digital intervention can produce large datasets containing information collected in a variety of formats. It may be necessary to collate relevant data across the datasets and compute new variables before usage analyses can be conducted. To simplify this process, Stage 1 proposes a checklist (see Multimedia Appendix 1) comprising a set of generic questions that will support a comprehensive understanding of the structure, processes, and content of the intervention in relation to data capture, contents of the datasheets, and factors related to trial implementation (eg, participant recruitment, Stage 1, Item 3). When used during the development phase of an intervention, the framework provides the opportunity to record and measure usage data that align with the proposed analysis plan. Ensuring efficient data capture at the outset can remove the need for extensive data cleaning and manipulation. When used for post hoc analysis only, Stage 1 can support the identification of appropriate usage variables and inform subsequent data cleaning and manipulation in preparation for analysis.

The usage data has been grouped into three categories. *Intervention characteristics* describes architecture, content, and expected workflow through the intervention, including intended usage [15] (eg, anticipated number of log-ins, number of available content components, and number of pages within a tunneled section). *Accrued data* covers all data collected during the running of the intervention, such as logs of interactions or log data (eg, date and time of use, pages viewed, and time spent on them) and user-entered data (eg, self-report). *Contextual data* encompasses previous findings related to the intervention development and trial (eg, factors affecting usage) and relevant external factors (eg, national health promotion campaigns).

*Accrued data* is usually collected automatically and recorded in datasheets, making the variables easier to extract and analyze. While some variables for intervention characteristics and contextual data may also be captured in this way (eg, number of log-ins), it is anticipated that additional measures may be either conceptual or external, meaning that they would not automatically be recorded as analyzable measures. For example, a digital intervention designed to increase physical activity may include pages addressing barriers to the target behavior, such as not having enough time to exercise or not having access to equipment. Pages containing advice to overcome these barriers may be distributed throughout the intervention, but are based on the same underlying theoretical concept (ie, an intervention characteristic). Therefore, a new variable needs to be created capturing usage of these pages and exposure to the theoretical underpinning. By identifying these measures of usage in advance, it is possible for additional data capture processes to be created, aiding efficient analyses after data collection. For example, depending on the delivery platform, code may be added to an intervention so that users who view these pages are recorded within a unique variable column in the log data. Contextual data is also less likely to be automatically collected and recorded within the study. If a large-scale outbreak of a respiratory infection occurs during the trial of an intervention aimed at reducing transmission of such infections, one might want to assess the potential impact on usage data. As with intervention characteristics, it may be possible to add further measures in order to capture personal experience of the illness or impact in a broader context.

It should be noted that the range of data available for collection may differ depending on the software used to develop and/or deliver the digital intervention. Using the framework during development to determine in advance which data are crucial may facilitate software development or else alternative workarounds. For example, where software is unable to collate total time spent on selected intervention pages, it will be necessary to ensure time spent by page is readily available to collate this after data collection. Interventions will also vary greatly in architecture and structure, depending on design, software, and delivery platform used (eg, website, app, or text based). For example, a *session* (Stage 1, Item 1) may refer to a single log-in, a component of content available across multiple log-ins, or the amount of times a specific activity is accessed. The framework provides a structure broad enough to be applied to different interventions. However, it is not anticipated that all criteria in the checklists will apply to every digital intervention. Where concepts and examples provided do not directly translate, researchers are encouraged to define them as relevant for their intervention and adapt the framework as needed.

### Stage 2: Selecting Measures of Usage and Generating Research Questions for Engagement

#### Overview

Establishing testable hypotheses is the precursor to carrying out systematic analyses. The aim of Stage 2 is to support the generation of specific research questions to drive testing of hypotheses. Stage 2 is divided into three sections to reflect the increasing complexity of comprehensive usage analyses: Section 1 helps define specific measures of usage (ie, descriptive statistics), while Sections 2 and 3 generate research questions (ie, bivariate and multivariate analyses).

#### Stage 2, Section 1: Descriptions of Usage Variables

The first section of Stage 2 provides a nonexhaustive list of potential usage measures. Example questions on the checklist (see Multimedia Appendix 2) demonstrate how measures of usage may be constructed.

The abundance of data and potential usage variables can encourage unsystematic data dredging. Identifying and reviewing the range of usage measures available enables researchers to make informed and/or theoretically driven decisions about what will be the most meaningful variables to include in any subsequent analysis plan. The process of familiarization with intervention content and architecture (Stage 1) may highlight considerations when selecting usage variables for analysis. For example, it may become apparent that certain sections of the digital intervention were considered to be of greater importance during the planning process, such as components that are theoretically informed (eg, pages containing BCTs or advice and goal-setting sections). Therefore, analyzing the usage of these pages, specifically, would be more meaningful than analyzing the total number of pages viewed. Alternatively, the intervention logic model may indicate that two theoretically based components are considered to have equal importance, yet they may have differing amounts of content within them, meaning that users would spend more time on one than the other. On that basis, analyzing the time spent on theory-based components may result in misleading conclusions about the impact of usage on health outcomes. In this scenario, a categorical usage metric may be more meaningful (eg, having completed or revisited the component). Analyzing a single usage measure is unlikely to provide a comprehensive understanding of engagement for all users across an intervention. However, combining multiple usage measures in a systematic way will provide a more detailed understanding of how users engaged with the intervention and what patterns of usage are associated with intended outcomes.

During intervention development, this process is undertaken prior to data collection and is therefore based on prospective data identified in Stage 1—bracketed numbers provided in Stage 2, Section 1, indicate their counterpart sections in Stage 1. Given the considerable crossover, we anticipate that these sections will be completed iteratively (see Figure 1). The purpose of Stage 1 is to identify all potential measures of usage available within the data; Stage 2 then narrows down that selection by considering which measures will provide the most informative understanding of usage for a specific intervention. The selection is informed by fundamental elements of the intervention highlighted in the planning process [13,14]. For example, in a digital intervention targeting weight loss, important measures of usage might be identified as entering weekly self-reports of weight, repeat use of recipe component, and time spent watching exercise videos. When carrying out post hoc analyses, descriptive statistics for measures of usage identified in Stage 2 may provide greater insight into which measures will be more informative (see Figure 1).

#### Stage 2, Sections 2 and 3: Relationships Between Usage, Participant Characteristics, Target Behaviors, and Behavioral Determinants

The remaining two sections of Stage 2 (see Multimedia Appendix 2) will guide the generation of specific research questions to assess how usage might be related to participant characteristics, behavioral determinants, and target behavior. While this stage can be used to generate limitless questions to drive exploration of the data, the framework is instead intended to be used to help select the most important questions that will answer theory-driven hypotheses. Usage variables are considered in relation to participant characteristics (Stage 2, Item 2), target behavior and behavioral determinants (Stage 2, Item 3), and behavior change across the intervention (Stage 2, Item 3). By answering these questions, it is anticipated that patterns of usage that reflect effective engagement with a specific intervention can be described. The moderating effect of demographic, psychosocial, and health factors (Stage 2, Item 2) on the relationship between usage and outcomes are also considered in Section 3 of Stage 2. When defining these variables, it is intended that the framework be adapted to individual interventions; for example, it is possible that a measure of usage (eg, uploading ongoing health monitoring statistics) may also be the intended primary outcome [13].

### Stage 3: Preparation for Analysis

The Stage 3 checklist (see Multimedia Appendix 3) supports the process of selecting appropriate types of analyses and analytical software, as well as the data preparation necessary to translate the research questions developed in Stage 2 into a plan of analysis. Generic questions guide the researcher to consider broad issues, such as available resources (Stage 3, Item 1) (eg, timeframe, additional researcher support, and analysis plan for efficacy), more specific issues of selecting appropriate type of analysis and analytical software (Stage 3, Item 2), and data management (Stage 3, Item 3) (eg, amalgamation, manipulation, and cleaning).

Our experience suggests that traditional statistical methods are not always suitable for analyzing the types of research questions generated by the framework. For example, while research is usually powered to analyze efficacy, it is frequently underpowered for the type of subgroup analyses needed for in-depth usage analyses. In addition, whereas analyses of amounts of usage (eg, total time spent or number of log-ins) often lend themselves to traditional methods, examining patterns of usage (eg, movement through pages) requires alternative methods to identify and inform subsequent statistical analysis. Therefore, techniques such as visualization and process mining may be more informative as they can reveal patterns of usage within the data, such as workflow through an intervention, clustering by participant groups, and temporal details [12,28-31]. In applying Stage 3 of the framework, the required data format for any analytical software should be considered.

## Application of the Analyzing and Measuring Usage and Engagement Data (AMUsED) Framework: Two Case Studies

### Overview

The following section provides researchers with practical examples of how the framework checklists can be applied in advance of or after data collection. The key findings and applications from utilizing the framework are highlighted below. The framework is necessarily comprehensive and completion of the checklists creates a lot of data and information. Indeed, this is the very process by which it supports the generation of systematic and rigorous usage analyses. The completed checklists for both studies have been amalgamated to enable comparison and are available as supplementary data (see Multimedia Appendices 4-6) so researchers using the framework in practice have detailed examples of its use.

### Applying the Framework During Development: Germ Defence

#### Overview

*PRImary care trial of a website-based Infection control intervention to Modify Influenza-like illness and respiratory infection Transmission* (PRIMIT) was a large randomized controlled trial (RCT) that showed a digital intervention to be effective at lowering the transmission of colds, influenza, and stomach upsets within the home through increased handwashing [32]. The framework was used to inform and structure the process of updating and amending the intervention to make it ready for dissemination as an open-access resource for use by the general public. As part of that process, the intervention was renamed *Germ Defence*. The research team involved with the dissemination was already familiar with the intervention, having worked on the design and evaluation of the PRIMIT study—for full details of the PRIMIT intervention and evaluation trial, please see Little et al [32].

#### Stage 1: Familiarization With the Data

Applying Stage 1 of the framework supported us in undertaking a detailed review of the original version of Germ Defence (Stage 1, Item 3.2; see Multimedia Appendix 4), along with data collected from the prior RCT (Stage 1, Item 2). This informed crucial updates to the collection of usage data and the generation of research questions, which we describe in the following sections.

Disseminating Germ Defence to the general public required us to strike a balance between obtaining informed consent to collect a minimal amount of data to support evaluation, while still enabling easy access to key aspects of the intervention by users who may be less willing to engage with standard research procedures (Stage 1, Item 3.1). Completing the Stage 1 checklist also allowed us to identify the following: (1) how the intervention and consent procedures should be streamlined (Stage 1, Items 3.1 and 3.2) and (2) what pertinent self-report data should be collected to enrich analyses of the automatically collected usage data and enable comparison with the prior RCT data (Stage 1, Item 1.2).

#### Stage 2, Section 1: Descriptions of Usage Variables

We reviewed the range of possible usage variables and identified which ones would provide the most informative picture of how Germ Defence was accessed and used during dissemination (see Multimedia Appendix 5). For example, the first component of the intervention contains compulsory tunneled pages, including a section for selecting handwashing goals. Examining dropout across this component and online consent pages, along with repeat use of the goal-setting section, will enable us to understand if and where users disengaged with the intervention. We then compared our list with the data collected from the prior RCT. This identified crucial amendments to the data capture process for Germ Defence that would otherwise have been missed. Specifically, data recorded on use of the goal-setting component was overwritten when revisited, losing both user-entered data and our ability to view movement backward and forward through these pages (Stage 1, Item 1.2). Identifying this issue in advance meant we were able to adapt the back-end processes to ensure the required data were captured.

#### Stage 2, Sections 2 and 3: Relationships Between Usage, Participant Characteristics, Target Behaviors, and Behavioral Determinants

Completion of Stages 1 and 2 of the framework in parallel helped us to narrow down our selection of usage-related questions to focus on behavioral determinants that were identified to be most strongly correlated with the target behavior in the prior RCT (see Multimedia Appendix 5). Since efficacy of Germ Defence has already been established from the prior RCT, the primary focus of the dissemination phase is to examine patterns of usage “in the wild” and their relationship to baseline user characteristics. The following research questions are a selection of those generated (Stage 2, Item 2):

1. Which pages see the highest amount of dropout, including consent and baseline measures?
2. How do users move through the goal-setting pages and what goals do they select?
3. Are baseline measures for handwashing, level of belief that handwashing will lower infection transmission, and/or belief in the ability to increase handwashing associated with usage?
4. Do users’ perceptions about the risk of infection to themselves or a household member relate to usage?
5. Do the means through which users hear about the website relate to usage?

Self-report data on behavioral and psychological variables will be collected using an optional survey (Stage 2, Item 3) in order to minimize potential dropout. This could be subject to selection bias with significant differences in the characteristics of users choosing to complete or not complete the survey. Any analysis examining the association between usage and behavioral outcome or change in behavioral determinants will be undertaken with caution. However, accessing or completing the survey may be operationalized as a measure of usage (Stage 2, Item 1), providing the opportunity to analyze relationships between intervention and survey usage (eg, Is viewing more intervention pages associated with completing the survey?) (Stage 2, Item 2). A comparison of baseline characteristics will also enable a check of whether those who complete the follow-up survey are different to those who do not.

#### Stage 3: Preparation for Analysis

The analytical tools available are SPSS for Windows version 24 (IBM Corp) and LifeGuide Visualisation Tool (University of Southampton) (Stage 3, Item 2; see Multimedia Appendix 6) [28,33]. It is anticipated that there will be insufficient power for definitive hypothesis testing. Patterns of usage (eg, repeat use and dropout across tunneled pages) will be best explored, initially, using visual tools. As Germ Defence has been built using LifeGuide software, the data produced will be compatible with the visualization tool (Stage 3, Item 3). Usage data collected from the intervention will need to be amalgamated and linked with self-report data from the optional survey. Thus, it was necessary to ensure that all users were allocated a unique nonidentifiable numeric ID upon first access so that all data can be linked (Stage 3, Item 3).

Using the research questions developed in Stage 2 and considerations highlighted in Stage 3, a full plan of analysis for Germ Defence was developed to inform efficient and systematic analysis after data collection. Applying the framework helped us prioritize research questions most relevant for the focus of the research (eg, how interventions are accessed and used “in the wild”) that would not be undermined by the constraints of using optional self-report measures.

### Applying the Framework for Post Hoc Analysis: Internet Dr

#### Overview

The framework was used to develop an analysis plan for usage data collected during an RCT of *Internet Dr*, a digital intervention to support the self-care of respiratory tract infections (RTIs) and to reduce unnecessary general practitioner (GP) visits. The RCT showed that users with access to Internet Dr were less likely to contact their GP about an RTI than those without access [32]. The usage analyses for Internet Dr will be conducted by researchers who were not involved in the original design, development, and evaluation of the intervention. The framework enabled the researchers to understand the intervention and associated data collection and to construct systematic research questions to investigate usage—for full details of the Internet Dr intervention and evaluation trial, please see Little et al [34].

#### Stage 1: Familiarization With Data

Internet Dr is structured around three components of theoretically based content. *Doctor’s Questions* and *Common Questions* aim to support users who are unsure if their symptoms are serious and whether they are in need of medical treatment (Stage 1, Items 1.2 and 3.2) [35]. *Treatment Options* is intended to increase self-efficacy for users who wish to manage symptoms they are finding distressing (Stage 1, Items 1.2 and 3.2) [36]. Applying Stage 1 aided understanding of how these three components relate to the psychological theories underpinning the intervention and, thus, the proposed determinants of the target behavior (ie, illness perception, health locus of control, willingness to tolerate symptoms, and treatment preferences; see Multimedia Appendix 4).

All content was available whenever users accessed the intervention across a 24-week period in the winter (Stage 1, Item 1.1). However, users were encouraged to log in specifically during periods of illness to help manage their symptoms. Completing the checklist emphasized the importance of recognizing these two distinct purposes for accessing the intervention: (1) to view content while ill and (2) to view content when well, perhaps out of curiosity. These differences in motivation to access the intervention when well or unwell may also be reflected in differences in patterns of usage.

#### Stage 2, Section 1: Descriptions of Usage Variables

Given the theoretically based content of the three components within the intervention, usage of each was identified as relevant to understanding underlying mechanisms of action (eg, number of users, number of pages viewed, time spent, and number of revisits) (see Multimedia Appendix 5). For the *Doctor’s Questions* component, compulsory tunneled pages are completed, leading to illness management advice on the last page (Stage 1, Item 1.1). Therefore, users of this component would, in theory, not benefit unless they had reached the final page, so completion and dropout were identified as important measures of usage for this component. It was also intended by design that users would view *Doctor’s Questions* first (Stage 1, Item 1.2). Thus, analyzing the order in which users visited the different components was important to understand whether the intervention was used as intended, as well as how intended versus nonintended order of use was related to users’ perceptions of their RTI, their perceived ability to self-manage, and whether they contacted their GP. As differences in users’ motivations for accessing the intervention may lead to differences in usage patterns (Stage 1), measures of usage identified in this component (eg, number of pages viewed and time spent) should be described for three situations: usage when ill, usage when well, and across all usage.

#### Stage 2, Sections 2 and 3: Relationships Between Usage, Participant Characteristics, Target Behaviors, and Behavioral Determinants

Considerations from the previous sections helped form pertinent research questions for the remaining two sections of Stage 2 (see Multimedia Appendix 5). For example, relationships between viewing specific content and theoretical constructs and behavioral determinants will be examined. As motivations for use have been identified as potentially influential, associations between reasons for accessing the intervention and patterns of usage and/or personal characteristics will be explored (Stage 2, Item 2). In addition, we will examine whether users followed the intended navigational paths and whether this was related to visiting their GP (Stage 2, Item 3). Below are some example research questions from the usage analysis plan:

1. Does usage of *Doctor’s Questions* differ when intervention access is made when ill compared to when well (eg, starting the component, number of pages viewed, time spent, and viewing the advice page)?
2. Are baseline personal characteristics associated with intervention use when ill or not ill?
3. Is viewing content during illness associated with lower GP visits?

Finally, we aim to identify whether viewing a specific piece or amount of content at a certain time (eg, when ill) led to a user being less likely to contact their GP.

#### Stage 3: Preparation for Analysis

SPSS for Windows version 24 (IBM Corp) and LifeGuide Visualisation Tool (University of Southampton) [21,26] will be used for data analysis (Stage 3, Item 2; see Multimedia Appendix 6). There will be sufficient power to analyze average usage of the intervention (eg, by whole sample) and associations with behavioral determinants and target behavior. Subgroup analyses are unlikely to be sufficiently powered (eg, comparing usage and outcomes of users accessing content when ill versus when prompted by completion of interim study measures). Some of the identified patterns of usage include movement through the intervention (Stage 3, Item 1) and will therefore be best explored visually (eg, the order in which the three content components were accessed). The datasheets are compatible with LifeGuide Visualisation Tool (University of Southampton). However, data is spread across several datasheets, requiring extraction, transformation, and amalgamation prior to analysis (Stage 3, Item 3).

Through completion of the three stages of the framework (see Multimedia Appendices 4-6), it was possible to break down a complex digital intervention and develop a comprehensive usage analysis plan, which will help identify what type of usage was successful in supporting self-management, for whom was it most beneficial, and at what time it was most influential.

### Comparing Case Studies

Both interventions target behavior associated with RTIs. However, Germ Defence focuses on infection prevention and may be accessed at any time, whereas Internet Dr supports self-management of symptoms while infected with an RTI. Despite the differences in their architecture, content, and function, the framework was suitably generic to be applied to both interventions. Although the same Stage 1 checklist was applied to both interventions, it enabled two completely different processes: for Germ Defence, Stage 1 helped shape structural changes to the intervention and data capture processes; for Internet Dr, Stage 1 enabled understanding of a previously unfamiliar and complex intervention and the accompanying datasheets (see Multimedia Appendix 4). Despite these different requirements, the checklist was comprehensive enough to fulfill both needs and lead to greater insights, such as realizing that a key component of Germ Defence (ie, the goal-setting component) was not capturing data as required, and understanding that Internet Dr was designed to be used during illness, but could be accessed at any time. Completing this first stage was the most complex and time consuming of the three stages for both interventions. However, through the thorough understanding of the intervention gained from Stage 1, the subsequent stages were easier to complete as the information was readily available to fit the generic questions. For example, having identified the theoretical underpinning of the three components of Internet Dr, their related measures of behavioral determinants, and expected relationship to GP contact, generating research questions to examine how usage related to changes in behavior and behavioral determinants was both simple and quick.

Through completing the checklist for Stage 2, Section 1, it is apparent that operationalizing usage in terms of amount was valid for both interventions (eg, number of log-ins, number of pages viewed, and time spent on pages; see Multimedia Appendix 5). This suggests that describing the extent to which an intervention has been used is a necessary first step for examining usage and that the number of pages viewed and time spent on them may inform our understanding of different styles of engagement. For example, spending more time on or revisiting pages may be indicative of higher levels of interaction compared to viewing pages briefly. However, as previously discussed, focusing on broad-based, summative descriptions of usage alone may not be sufficient to understand how the intervention supported change in target behaviors and outcomes. For example, distinguishing and comparing usage of the three theoretically based content components of Internet Dr will aid understanding of the potential mechanisms of action within the intervention.

The differences in research questions generated from Stage 2 highlight the differences in structure between the two interventions. Germ Defence is a stand-alone intervention requiring access only once, with an optional follow-up survey. Once completed, the data generated from using the intervention will provide a snapshot of behavior at that time. On that basis, research questions focus on user characteristics and behavioral determinates and target behavior at baseline (see Multimedia Appendix 5). In contrast, in addition to self-report and log data over a 6-month period, Internet Dr users’ GP notes providing information for the year before and after the trial commenced were also collected. This depth and length of duration of data collection enables different research questions, including consideration of behavior prior to the trial and for some months after.

Stage 2 of the framework highlighted the relevance of examining patterns of usage (eg, movement through the intervention) and subgroups usage analyses. As both interventions have insufficient power to analyze subgroup usage, and patterns of usage lend themselves to visual exploration (see Multimedia Appendix 6), this supports the use of contemporary visualization tools in addition to traditional statistical methods.

Both interventions include research questions examining relationships between participant characteristics and usage and whether any of those characteristics moderate the relationship between usage and target behavior. Through comparison of these similar analyses across multiple interventions, it will become possible to build up a pattern of how personal characteristics may influence digital intervention usage, leading to generic learning points to inform future intervention design. This may also be the case for usage analyses of interventions with similar aims (eg, self-management of illness) or similar theoretical underpinning, behavioral determinants, or BCTs. Once a body of research is assembled, it would be possible to use the framework in advance to structure data capture and analysis so that it is comparable with prior interventions and published research.

## Discussion

### Overview

The AMUsED framework aims to support detailed and systematic analysis of digital intervention usage. The framework comprises three stages of checklists for researchers to do the following: (1) understand the intervention’s design, theoretical underpinning, and data collection processes; (2) define meaningful variables to assess usage and generate both broad and fine-grained research questions to examine relationships between usage, participant characteristics, and target behavior and behavioral determinants; and (3) prepare datasheets and consider appropriate software for analysis.

The framework has been applied to two digital interventions: Germ Defence promotes RTI prevention and Internet Dr supports self-management of RTI symptoms. Using the framework while preparing Germ Defence for public dissemination identified necessary amendments to data capture processes. For Internet Dr, the framework helped guide a research team who were previously unfamiliar with the intervention design and data to devise a comprehensive usage analysis plan. The case studies demonstrate the flexibility of the framework to be applied to different interventions and the advantages of using the framework, both before and after data collection.

### Implications

The AMUsED framework checklists provide researchers with easily applied templates for carrying out detailed usage analyses of digital interventions. The framework supports the level of rigor in reporting digital intervention content and findings called for by current guidelines from the UK Medical Research Council, the Consolidated Standards of Reporting Trials of Electronic and Mobile HEalth Applications and onLine TeleHealth (CONSORT-EHEALTH), and the Template for Intervention Description and Replication (TIDieR) [22,23,27,37]. The checklists extend upon and draw together previous work on categorizing digital intervention usage data and selecting research questions [19-21,24,25] by providing a systematic and comprehensive process for researchers to follow. The process can be incorporated into existing digital intervention development methods, such as the person-based approach [14] and the behavioral intervention technology model [15], enabling pretesting of data capture processes to support theory-based hypothesis testing.

The framework encourages usage analyses that will broaden our understanding of mechanisms of action underlying a specific digital intervention, explaining the relationships between user characteristics, patterns of usage, and behavior change. Through this process, it may be possible to identify effective engagement, finding the level of usage necessary for a specific intervention in order to change the target behavior [11]. This will lead to digital interventions being developed to be more concise, efficient, and targeted, making them less arduous for the user and supporting higher rates of uptake and engagement.

### Limitations

The framework has been developed and tested using Web-based interventions built using the same software [33] that captures extensive log data and has the ability for researchers to write additional code in order to capture tailor-made usage measures. Digital interventions may be delivered across a wide variety of platforms (eg, text messaging, apps, and websites) and developed using different software. This leads to substantial variation in design, the manner in which they are written or coded, and the availability and format of data collected. The framework is flexible enough to be applied across diverse interventions and sufficiently detailed to generate specific testable hypotheses for most digital interventions. However, we welcome other researchers to use the framework and build upon it based on their experience.

The AMUsED framework focuses on the analysis of measures of usage as one facet of engagement. Where objective measures of physiological reactions (eg, cardiac activity and eye tracking) or subjective self-report measures of engagement are available, it is hoped that future research may examine these alongside usage data and develop the framework further to incorporate them, thereby increasing our ability to explain not just the role of usage, but engagement more broadly [6]. It is also our hope that the framework will be applied as part of a mixed-methods approach, triangulating usage analyses with insights and experiences collected qualitatively [6,8,10].

The framework has been applied to ensure adequate data collection when used during the development phase of digital interventions. However, the framework also has the potential to be used to inform study design to answer empirical questions on effective engagement; for example, multiphase optimization strategy (MOST) and sequential multiple assignment randomized trial (SMART) [38]. Although this paper does not address this application, it provides an avenue for future research for the wider application of the framework.

### Conclusions

The AMUsED framework offers a systematic process for carrying out in-depth usage analyses. The aim of the framework is to capture and formalize the techniques used by experienced researchers to support researchers who are new to conducting usage analyses, or new to a particular intervention, in deciding how to assess usage data that will be or has been collected. Using the framework will benefit researchers by lowering the possibility of overlooking key questions and making the reporting of usage analyses more efficient, leading to a quicker turnaround for publishing. The checklists provide the means to increase transparency and make findings easier to replicate, while discouraging unsystematic data dredging. The process will also encourage greater detail and consistency in the reporting of usage and engagement, making it easier to apply the findings to a wider context [7] and enabling comparison across different interventions and evaluation studies. The framework helps to operationalize and measure usage in ways that will better inform our understanding of engagement with a digital intervention, encompassing broad measures of usage by the whole sample, through to specific theory-based usage variables and usage by subgroups based on personal characteristics. It guides insight into which components of an intervention worked and how they interacted with users’ personal characteristics. Finally, by using the framework it may be possible to identify the extent of usage required to support changes in behavior and health-related outcomes and, thus, an understanding of what constitutes an effective level of engagement for specific interventions.

## Appendix

Multimedia Appendix 1 Stage 1 checklist for the Analyzing and Measuring Usage and Engagement Data (AMUsED) framework.

Multimedia Appendix 2 Stage 2 checklist for the Analyzing and Measuring Usage and Engagement Data (AMUsED) framework.

Multimedia Appendix 3 Stage 3 checklist for the Analyzing and Measuring Usage and Engagement Data (AMUsED) framework.

Multimedia Appendix 4 Stage 1 checklist with case studies.

Multimedia Appendix 5 Stage 2 checklist with case studies.

Multimedia Appendix 6 Stage 3 checklist with case studies.

## Abbreviations

AMUsED: Analyzing and Measuring Usage and Engagement Data
BCT: behavior change technique
CONSORT-EHEALTH: Consolidated Standards of Reporting Trials of Electronic and Mobile HEalth Applications and onLine TeleHealth
GP: general practitioner
MOST: multiphase optimization strategy
PRIMIT: PRImary care trial of a website-based Infection control intervention to Modify Influenza-like illness and respiratory infection Transmission
RCT: randomized controlled trial
RTI: respiratory tract infection
SMART: sequential multiple assignment randomized trial
TIDieR: Template for Intervention Description and Replication
