# Supplementary material for: A Framework for Analyzing and Measuring Usage and Engagement Data (AMUsED) in Digital Interventions: Viewpoint
Source: J Med Internet Res. 2019 Feb 15;21(2):e10966. doi: 10.2196/10966 (PMC6396072; doi:10.2196/10966)
Supplement: Multimedia Appendix 1 [file jmir_v21i2e10966_app1.pdf]

Stage 1 checklist for the Analyzing and Measuring Usage and Engagement Data (AMUsED) framework

Familiarisation with the data – identifying variables

Generic questions by data type	Intervention Name:
**1. Intervention characteristics. Data for intervention architecture and content.**	
**1.1. Workflow. Intervention structure and expected participant interaction and navigation through the intervention.**	
How many logins/sessions are available?	
When are they available?	
Are new sessions released depending on time elapsed or task-completion?	
Are there limitations on the availability of the intervention?	
Is the purpose of a session to collect self-report measures and/or use the intervention?	
When is the intervention considered to be finished?	
What prompts are used to encourage usage (e.g. emails, texts, notifications) and when are they sent?	
Does the intervention contain 'tunneled' (compulsory) sequences of pages which users have to view to move forward?	
Are users able to select linked components they wish to view, and avoid others?	
**1.2. Content. Content available within the pages of the intervention.**	
What are the components available?	
What is the aim of each component and are they based on underlying theoretical constructs?	
In what order is it anticipated the components will be used?	
What interactive features are available (e.g. forums, videos, printable information)? How long should they take to complete?	
Are all components/features available to all users throughout the intervention or are some tailored for specific times or users?	
Which pages are for collecting self-report measures or for administrative purposes (e.g. questionnaires, login, password change)?	
Are there specific pages to mark the start and end of sessions?	
Which pages contain BCTs (e.g. information, planning, feedback) and what are they?	
In which sessions are they available?	

Can specific BCTs be identified on particular pages or groups of pages? How many groups are there?	
Do any of the pages have response options to collect information in addition to baseline/follow-up measures? What data is collected?	
**2. Accrued data. Data collected during an intervention.**	
**2.1. Self-report. Users' self-reported responses collected across various stages of the trial.**	
When are self-report questionnaires collected (e.g. weekly logins, monthly symptom information, follow-up at 6 months)?	
What demographic information is available (e.g. age, gender, education)?	
Which measures are specifically related to the target behavior and how often are they collected?	
Which measures of beliefs influential on the target behavior are collected and when?	
Are measures of health collected (e.g. conditions which may impact on target behavior or are co-morbid) and psychosocial factors (e.g. anxiety, illness perception, motivation)?	
Are additional measures collected at follow-up (e.g. satisfaction, adherence)?	
**2.2. Log-data. Information automatically collected through engagement with an intervention.**	
What data is the software platform able to record?	
Are number, date and time of logins available by individual user?	
Are individuals' total durations of usage accessible?	
Are the number and time of usage prompts recorded?	
Are there details for which pages were viewed, the sequential order and time spent viewing?	
**2.3. External data. Data collected independently but alongside intervention usage.**	
How and where is the data collected (e.g. GP or support staff notes, lab reports, other digital data such as activity or location trackers)?	
What data is collected?	
Which of these measures relate to or may impact on the target behavior?	
**3. Contextual data. Data indirectly related to the running of the intervention which may be influential over usage and analysis.**	
**3.1. External factors. Structures and events which may influence participation in the intervention.**	
How are users recruited to the intervention?	
Did any specific large-scale events, with the potential to impact on the intervention, occur during the period of the intervention (e.g. changes in	

treatment, health campaigns, illness outbreak, technical issues with the intervention)?	
**3.2. Previous theory and findings. Results of behavioral analyses carried out during intervention development (e.g. logic models), and analyses of clinical outcomes if available.**	
What are the hypothesized mechanisms of the intervention (e.g. as specified in the intervention's logic model)?	
Which factors are identified as important in qualitative research, and can they be related to the variables collected in the trial (e.g. preferences for specific pages)?	
Which variables are identified as relating to outcomes (e.g. behavioral determinants, theoretical constructs, health factors)?	
