# Supplementary material for: A Framework for Analyzing and Measuring Usage and Engagement Data (AMUsED) in Digital Interventions: Viewpoint
Source: J Med Internet Res. 2019 Feb 15;21(2):e10966. doi: 10.2196/10966 (PMC6396072; doi:10.2196/10966)
Supplement: Multimedia Appendix 2 [file jmir_v21i2e10966_app2.pdf]

Stage 2 checklist for the Analyzing and Measuring Usage and Engagement Data (AMUsED) framework

## Selecting usage variables and generating research questions

| Generic questions | Intervention Name: |
|---|---|
| **1. Descriptions of usage variables. Which usage variables are relevant to the intervention and in which format (e.g. number of users/sessions, duration, percentage of total, dichotomous)?** | |
| Completing intervention/trial period (stage1; 1.1 & 2.2). E.g. How many users complete the trial? What is the average time taken to complete? | |
| Logins or sessions where the intervention was accessed (stage 1; 1.1 & 2.2). E.g. How many users start/complete each login/session? How long does it take to complete each session? How many pages are viewed within the session? Which session has the highest proportion of pages viewed, or duration of time spent on it? | |
| Date of login and usage. E.g. When do users login? What time of year? Are there changes in frequency of logins? | |
| Time of day of login and usage. E.g. What time of day is usage? Are users more likely to spend longer on the intervention at certain times? | |
| Days/weeks of usage (stage1; 1.1 & 2.2). E.g. For how many days/weeks out of the total is the intervention accessed for? How many times within a week is the intervention accessed? Are there repeated uses within the same day? | |
| Response to prompts/notifications (e.g. requests to login, email, text, upload data) (stage1; 1.1 & 2.2). E.g. How many responses are sent? How long after receiving notification do users take to log-in or respond? | |
| Features/linked menu components used (stage 1; 1.1, 1.2 & 2.2). E.g. How many features/components are accessed? How many users access each one? Which are completed and by how many users? Which feature/component has the highest proportion of pages viewed or time spent? What order are they viewed in? Is this the anticipated order? Which have the highest proportion of drop-out? | |
| Revisiting components/features (stage 1; 1.1, 1.2 & 2.2). E.g. Are any used repeatedly? How many times are they revisited, and for how long? Which are most revisited? | |
| Type of content/BCTs used (excluding administration pages) (stage 1; 1.2 & 2.2). E.g. How many groups of pages with similar content are accessed and | |

| | |
|---|---|
| by how many users? How many pages within the group are used? How many users view each page? Which groups of pages have the highest proportion of views? Which pages are viewed at each login, and when is the largest amount of pages viewed? Which pages have higher drop-out? | |
| Completing ongoing measures (e.g. monthly questionnaires, response options within content pages, uploading information or text responses) (stage 1; 2.1 & 2.2). E.g. How many users complete ongoing measures? When do they complete them? Do they also access the intervention at that time? | |
| External device usage (e.g. wearables and other sensor technologies) (stage 1; 2.3). E.g. How much time is spent with the device? How many times is it used? What number of days/weeks is it used for? | |
| **2. Relationships between usage and participant characteristics. Are users' demographic, physical or psychosocial characteristics at baseline related to intervention usage?** | |
| Are any characteristics at baseline related to usage? E.g. Is anxiety associated with revisiting features? Is current health related to usage of external devices? Are users who spend more time on the intervention older than those who spend less time? Which characteristics are associated with drop-out? | |
| Are any contextual factors associated with usage (stage1; 3)? E.g. Is manner of recruitment related to usage? | |
| Do high/low users differ by other usage factors? E.g. Do users who spend more time on the intervention view more types of content than users who spend less time? Is usage of an external device related to intervention usage? | |
| **3. Relationships between usage, behavioral determinants, and target behaviors. Which usage variables are associated with follow-up measures for target behavior and behavioral determinants? Which usage variables help explain changes in behavior across the intervention?** | |
| Are baseline measures for behavioral determinants/target behavior related to usage? E.g. Is the number of days the intervention is used for related to a behavioral determinant? Do users with low target behavior spend less time on the intervention? | |
| Which usage variables are related to behavioral determinants/target behaviors and at follow-up? E.g. Do users who view a group of pages containing a specific BCT score higher/lower for the associated behavioral determinant? Is completing/not completing a particular component associated with target behavior at follow-up? Is the time spent on a session related to target behavior? | |
| Is usage associated with measures for acceptability/satisfaction at follow-up? | |

| | |
|---|---|
| E.g. Are high levels of satisfaction associated with accessing more pages? Do users with low satisfaction spend less time using external devices? | |
| Do users who report positive changes in behavioral determinants/target behavior from baseline to follow-up use the intervention differently to those who do not? E.g. Do users who report positive increases in a behavioral determinant view more pages from a specific component containing an associated BCT? Do users who report positive behavior change spend more time on the intervention? | |
| Are relationships between usage and target behavior moderated by demographic, psychosocial or health factors? E.g. Is the relationship between time spent on the intervention and target behavior altered when moderated by anxiety? | |
| What level of usage is necessary for 'effective engagement'? E.g. Do outcome measures plateau after viewing certain content, or after a certain amount of time or sessions completed? | |
