# Supplementary material for: A Framework for Analyzing and Measuring Usage and Engagement Data (AMUsED) in Digital Interventions: Viewpoint
Source: J Med Internet Res. 2019 Feb 15;21(2):e10966. doi: 10.2196/10966 (PMC6396072; doi:10.2196/10966)
Supplement: Multimedia Appendix 3 [file jmir_v21i2e10966_app3.pdf]

Stage 3 checklist for the Analyzing and Measuring Usage and Engagement Data (AMUsED) framework

Preparation for analysis

Generic questions	Intervention Name:
**1. Resources**	
What is the timeframe for completing the analyses?	
What resources are needed? E.g. additional research time, expertise	
Is a plan of analysis already available? How does the analysis plan developed using the framework compare to that plan? Are changes or updates needed?	
Is ethical clearance in place to carry out usage analyses?	
**2. Selecting types of analysis and analytical software**	
Will the usage data be triangulated with qualitative data?	
What analytical tools are available?	
Is there sufficient statistical power to answer the planned research questions?	
Can the selected measures of usage be analyzed using the available tools? Is bespoke software necessary (e.g. visualisation techniques)?	
**3. Data preparation**	
When is the data available?	
Is the data raw or has it been used/cleaned previously?	
How many datasheets are there? Will these need to be amalgamated?	
Is the data structured to work with the tools available? What formats are the datasheets in (e.g. excel, .csv) and will they need converting for analysis?	
What preparation does the data need (e.g. cleaning, anonymizing)?	
Are all variables readily available or will they need extracting/transforming/recoding?	
Is the data in the right format to answer the research questions? Will it need adapting (e.g. continuous variables changed to categorical)?	
