# Supplementary material for: A Framework for Analyzing and Measuring Usage and Engagement Data (AMUsED) in Digital Interventions: Viewpoint
Source: J Med Internet Res. 2019 Feb 15;21(2):e10966. doi: 10.2196/10966 (PMC6396072; doi:10.2196/10966)
Supplement: Multimedia Appendix 4 [file jmir_v21i2e10966_app4.pdf]

Stage 1 checklist for the AMUsED Framework: PRIMIT Study and Internet Dr

Familiarisation with the data – identifying variables		
Generic questions by data type	Intervention: *PRIMIT*	Intervention: *Internet Dr*
**1. Intervention characteristics. Data for intervention architecture and content.**		
**1.1 Workflow. Intervention structure and expected participant interaction and navigation through the intervention.**		
How many logins/sessions are available?	*4 sessions. For dissemination: change to 1 session (see previous findings 3.2)*	*Not structured as sessions, access intended during an RTI.*
When are they available?	*1st at baseline, 2nd released 3.5 days post-baseline, 3rd at 10.5 days, 4th at 17.5 days post-baseline.*	*All content available at every access. Prompted logins at: baseline, 5 x 4-weekly interim questionnaires, & follow-up questionnaires. Then during illness and 48 hour follow-up.*
Are new sessions released depending on time elapsed or task-completion?	*Released after the times set, and dependent on having accessed previous session.*	*No.*
Are there limitations on the availability of the intervention?	*Previous session must be accessed in order to view next. Content from previous sessions could be revisited in subsequent session.*	*No.*
Is the purpose of a session to collect self-report measures and/or use the intervention?	*Session 1 includes baseline measures and content, all other sessions are content alone.*	*Questions are asked after login to ascertain user's purpose – either to use intervention for illness or to complete interim questionnaires.*
When is the intervention considered to be finished?	*At the end of the 4th session.*	*The study finishes after the follow-up questionnaire at 24 weeks. The intervention was finished when users no longer wished to access it.*
What prompts are used to encourage usage (e.g. emails, texts, notifications) and when are they sent?	*An email is sent when next session is ready, followed by 2 further emails if they don't login. For dissemination: 2 emails prompting login to follow-up survey.*	*Email prompts for 4 weekly data collection. Additional emails are sent after logging in during illness to prompt repeat login after 48 hours.*
Does the intervention contain 'tunneled' (compulsory) sequences of pages which users have to view to move forward?	*Initial pages of each session are tunneled. For dissemination: tunneled pages from session 1 will be at start of intervention.*	*'Doctor's Questions' component is tunneled as symptoms are assessed before providing illness management advice.*
Are users able to select linked components they wish to view, and avoid others?	*Optional menu components are available after completing tunneled pages. For dissemination: 3 menu components will be available using content from sessions 2-4.*	*There are 3 linked menu components available from the home page.*
**1.2. Content. Content available within the pages of the intervention.**		

What are the components available?	*Tunneled components in sessions 1-4 providing information on handwashing. Menu components: more advice on looking after someone with flu, details about the research, revisiting goal-setting, revisiting information from previous sessions. For dissemination: tunneled component from session 1. Menu components for more advice on handwashing, flu, and details about the research. There is also the opportunity to return to the start of the tunneled pages again.*	*'Doctor's Questions'. Asks about symptoms & provides advice recommending either: self-management, phoning NHS Direct, or seeking immediate medical attention. 'Treatment Options'. Advice on coping with symptoms: without medication, with medication, and boosting immune system. 'Common Questions'. 2 sets of FAQs: 'Ask the Internet Dr' - medical questions about illness and treatment, 'Common myths about Colds and Flu' - general questions about illness beliefs.*
What is the aim of each component and are they based on underlying theoretical constructs?	*Messages based on the theory of planned behaviour* [1] *promote handwashing as an effective behaviour (positive attitudes), socially desirable (subjective norms), and easy to do (perceived behavioral control). Messages utilizing protection motivation theory* [2] *provided information on health consequences and infection transmission for RTIs.*	*Doctor's Questions and Common Questions support users who are unsure if their symptoms are serious and whether they need medical treatment. Based on Leventhal's Common Sense Model of Self-regulation of health and illness* [3] *to increase understanding of illness. Treatment Options supports management of distressing symptoms. Based on Bandura's Social Cognitive Theory* [4] *to increase self-efficacy.*
In what order is it anticipated the components will be used?	*Tunneled components have to be used first. No expectation for menu components.*	*Doctor's Questions first so as to check symptoms.*
What interactive features are available (e.g. forums, videos, printable information)? How long should they take to complete?	*Goal setting component - users with low intended handwashing who select no intention to improve receive supportive messages encouraging them to review their choices. An optional print-out page is available.*	*Videos available: Welcome video on home page: 1 minute, 23 seconds, glands: 48 seconds, meningitis/septicaemia: 1 minute, 1 second, video sinusitis: 30 seconds. Printable material available but log-data doesn't record whether it was used.*
Are all components/features available to all users throughout the intervention or are some tailored for specific times or users?	*Users self-report amount of handwashing, efficacy and necessity beliefs at the start of sessions 2-4. Users with low handwashing and beliefs are presented with additional tailored information during the tunneled component. Some content is also tailored depending on household type (e.g. for users with children). For dissemination there will be no tailoring by household.*	*Doctor's Questions and Treatment Options are split into cough, sore throat, runny nose & fever.*
Which pages are for collecting self-report measures or for administrative purposes	*Page names starting with 's' or 't' indicate active content.*	*Separate list available for content by individual page.*

Are there specific pages to mark the start and end of sessions?	*First page of tunneled component marks start of each session.*	*Home page is the first page visited.*
Which pages contain BCTs (e.g. information, planning, feedback) and what are they?	*Tunneled pages at the start of each session contain: messages to increase perceived risk, information for explanation of infection transmission by hand, motivation to increase positive attitudes to handwashing, information on viral load and washing hands to reduce infection, goal setting. Tailored pages within tunneled components contain messages to support: habit formation, overcoming barriers, understanding necessity.*	*See components listed previously*
In which sessions are they available?	*Across all 4.*	*N/a*
Can specific BCTs be identified on particular pages or groups of pages? How many groups are there?	*Goal setting in session 1 provides cues and feedback over 4 pages. Tailored pages across sessions 2-4 are grouped by handwashing, necessity, and efficacy. Pages addressing attitudes, barriers and beliefs are used throughout the tailored pages.*	*See components listed previously*
Do any of the pages have response options to collect information in addition to baseline/follow-up measures? What data is collected?	*Goal setting pages collect actual and intended handwashing plan. However, repeat use within a session is not recorded, only the last entry is stored. For dissemination: amend to capture repeated page viewings and goal entries.*	*Information on symptoms is collected in Doctors Questions.*
**2. Accrued data. Data collected during an intervention.**		
**2.1. Self-report. Users' self-reported responses collected across various stages of the trial.**		
When are self-report questionnaires collected?	*Baseline and every 4 weeks up to 12 weeks. For dissemination: create optional follow-up survey completed one week later.*	*Baseline, every 4 weeks up to 24 weeks, during illness and 48 hour follow up.*
What demographic information is available (e.g. age, gender, education)?	*Baseline: Age, gender, qualification, household. For dissemination: add measure for how they heard about the intervention.*	*Baseline: Age, gender, qualification, household, smoker, alcohol, ethnicity.*
Which measures are specifically related to the target behaviour and how often are they collected?	*Baseline, session 2-4, 4 & 12 weeks: Actual and intended handwashing behaviour. Every 4 weeks: User and household illness occurrence. For dissemination: follow-up measures at 1 week for current and intended handwashing behaviour,*	*During illness, every 4 weeks & follow-up (see also external data): occurrence of illness and contact with all NHS.*

	necessity and efficacy.	
Which measures of target behavioral determinants are collected and when?	*Baseline, 4 & 12 weeks: TPB* [1] *intentions, attitudes, norms, beliefs.*	*Baseline & follow-up: health locus of control* [5], *Krantz health opinion survey* [6], *TPB* [1]. *When ill & 48hr: IPQ-R* [7], *TPB* [1].
Are measures of health collected (e.g. conditions which may impact on target behaviour or are co-morbid) and psychosocial factors (e.g. anxiety, illness perception, motivation)?	*Flu vaccination.*	*Baseline, when ill & 48 hour, & follow-up: (Physical) Mobility, self-care, usual activities, pain, anxiety/depression. Baseline (see also external data): health anxiety inventory* [8].
Are additional measures collected at follow-up (e.g. satisfaction, adherence)?	*Week 8: Satisfaction questions. For dissemination: acceptability e-scale* [9].	*Follow-up: patient enablement* [10], *website satisfaction, problematic experiences of therapy scale* [11].
**2.2. Log-data. Information automatically collected through engagement with an intervention.**		
What data is the software platform able to record?	*Time and date, pages viewed & order, time spent on pages, self-report measures.*	*Time and date, pages viewed & order, time spent on pages, self-report measures.*
Are number, date and time of logins available by individual user?	*Yes.*	*Yes.*
Are individuals' total durations of usage accessible?	*Needs to be extracted.*	*Needs to be extracted.*
Are the number and time of usage prompts recorded?	*Overall scheduled timings for emails are available, but not sent times by individual.*	*Overall scheduled timings for emails are available, but not sent times by individual.*
Are there details for which pages were viewed, the sequential order and time spent viewing?	*Yes.*	*Yes.*
**2.3. External data. Data collected independently but alongside intervention usage.**		
How and where is the data collected?	*Hand collected by the research team from users' GP notes.*	*Hand collected by the research team from users' GP notes.*
What data is collected?	*Visits to a GP for an RTI or gastrointestinal infection during trial period. Antibiotic prescriptions for RTI.*	*Number of GP visits for RTI during trial, for year prior to trial, and co-morbid illnesses. Antibiotic prescriptions for RTI.*
Which of these measures relate to or may impact on the target behaviour?	*Number of GP visits for RTI by user*	*Number of GP visits for RTI during trial, for year prior to trial, and co-morbid illnesses.*
**3. Contextual data. Data indirectly related to the running of the intervention which may be influential over usage and analysis.**		
**3.1. External factors. Structures and events which may influence participation in the intervention.**		
How are users recruited to the intervention?	*Recruited via GP using paper consent. For dissemination: move to online consent and capture where users heard about the intervention.*	*Recruited via GP.*
Did any specific large-scale events, with the	*Outbreak of swine flu prior to trial commencing.*	*N/a*

potential to impact on the intervention, occur during the period of the intervention?		
**3.2. Previous theory and findings. Results of behavioral analyses carried out during intervention development (e.g. logic models), and analyses of clinical outcomes if available.**		
What are the hypothesized mechanisms of the intervention (e.g. as specified in the intervention's logic model)?	*Handwashing will increase when intentions to wash hands are raised through positive attitudes, norms and beliefs. Increasing perceptions of risk from an RTI will lead to increased intentions to handwash.*	*Users with low understanding of their symptoms will be less likely to consult their GP having viewed Doctors Questions and Common Questions. Users with low self-efficacy in their ability to self-manage their illness will be less likely to consult their GP having viewed Treatment Options.*<br>*Level of anxiety may be associated with usage.*
Which factors are identified as important in qualitative research, and can they be related to the variables collected in the trial?	*N/a*	*N/a*
Which variables are identified as relating to outcomes (e.g. behavioral determinants, theoretical constructs, health factors)?	*The largest increase in handwashing occurred at the first session* [12]. *For dissemination: amend from 4 to 1 session. Perceived risk of infection was a key predictor of intentions to handwash* [12]. *For dissemination: capture risk at baseline.*	*Analysis of the data from the RCT focused on the outcomes of GP contacts and antibiotic use* [13]. *No analyses were carried out for usage, behavioral determinants or personal characteristics.*

1. Ajzen I. The theory of planned behavior. *Organ Behav Hum Decis Process*. 1991;50(2):179-211. doi:10.1016/0749-5978(91)90020-T

2. Rippetoe PA, Rogers RW. Effects of components of protection-motivation theory on adaptive and maladaptive coping with a health threat. *J Pers Soc Psychol*. 1987;52(3):596-604. PMID: 3572727

3. Leventhal HA, Brissette I, Leventhal EA. The common-sense model of self-regulation of health and illness. In: LD Cameron, HA Leventhal (Eds), *The self-regualtion of health and illness behavoiur*. London, UK: Routledge; 2003:42-65. ISBN: 9780415297011

4. Bandura A. *Self-Efficacy: The Exercise of Control*. New York, US: WH Freeman; 1997. ISBN: 9780716728504

5. Wallston KA. The validity of the multidimensional health locus of control scales. *J Health Psychol*. 2005;10(5):623-631. PMID: 16033784

6. Krantz DS, Baum A, Wideman MV. Assessment of Preferences for Self-Treatment and Information in Health Care. *J Pers Soc Psychol*. 1980;39(5):977-990. PMID: 7441487

7. Moss-Morris R, Weinman J, Petrie K, Horne R, Cameron L, Buick D. The Revised Illness Perception Questionnaire (IPQ-R). *Psychol Health*. 2002;17(1):1-16. doi:10.1080/08870440290001494.

8. Salkovskis PM, Rimes KA, Warwick HMC, Clark DM. The Health Anxiety Inventory: development and validation of scales for the measurement of health anxiety and hypochondriasis. *Psychol Med*. 2002;32(5):843-853. PMID: 12171378

9. Tariman JD, Berry DL, Halpenny B, Wolpin S, Schepp K. Validation and testing of the Acceptability E-scale for web-based patient-reported outcomes in cancer care. *Appl Nurs Res*. 2011;24(1):53-58. PMID: 20974066

10. Howie JG, Heaney DJ, Maxwell M, Walker JJ. A comparison of a Patient Enablement Instrument (PEI) against two established satisfaction scales as an outcome measure of primary care consultations. *Fam Pract*. 1998;15(2):165-171. PMID: 9613486

11. Kirby S, Donovan-Hall M, Yardley L. Measuring barriers to adherence: validation of the problematic experiences of therapy scale. *Disabil Rehabil*. 2014;36(22):1924-1929. PMID: 24410171

12. Ainsworth B, Steele M, Stuart B, et al. Using an Analysis of Behavior Change to Inform Effective Digital Intervention Design: How Did the PRIMIT Website Change Hand Hygiene Behavior Across 8993 Users? *Ann Behav Med*. December 2016:1-9. PMID: 27909944

13. Little P, Stuart B, Andreou P, et al. Primary care randomised controlled trial of a tailored interactive website for the self-management of respiratory infections (Internet Doctor). *BMJ Open*. 2016;6(4):e009769. PMID: 27098821
