# Supplementary material for: A Framework for Analyzing and Measuring Usage and Engagement Data (AMUsED) in Digital Interventions: Viewpoint
Source: J Med Internet Res. 2019 Feb 15;21(2):e10966. doi: 10.2196/10966 (PMC6396072; doi:10.2196/10966)
Supplement: Multimedia Appendix 5 [file jmir_v21i2e10966_app5.pdf]

Stage 2 checklist for the AMUsED Framework: PRIMIT Study and Internet Dr

Selecting usage variables and generating research questions		
Generic questions	Intervention: *Germ Defence*	Intervention: *Internet Dr*
**1. Descriptions of usage variables. Which usage variables are relevant to the intervention and in which format (e.g. number of users/sessions, duration, percentage of total, dichotomous)?**		
Completing intervention/trial period (stage 1; 1.1 & 2.2).	*Number of users completing tunneled component. Number of pages viewed (including baseline measures, tunneled and individual menu components). Time spent by page.*	*Number of users completing follow-up measures at 24 weeks. Number of users ill during trial period (logged in during illness, self-report at 4 week measures, from GP notes). Total number of pages viewed across the trial and pages with the greatest number of views.*<br>*Total time spent on pages.*
Logins or sessions where the intervention was accessed (stage 1; 1.1 & 2.2).	*N/a*	*Purpose of login: to complete baseline, 4 weekly measures or manage illness. Content viewed, number of pages and time spent when using intervention during illness.*
Date of login and usage.	*N/a*	*n/a*
Time of day of login and usage.	*N/a*	*Proportion of usage outside vs during GP hours.*
Days/weeks of usage (stage 1; 1.1 & 2.2).	*N/a*	*Number of times intervention content was accessed.*
Response to prompts/notifications (stage 1; 1.1 & 2.2).	*Number of users accessing the survey. Number of users completing the survey.*	*Number of users who logged in to illness follow-up at 48 hour.*
Features/menu components used (stage 1; 1.1, 1.2 & 2.2).	*Number of users viewing each menu component. Number of pages viewed in menu components. Number of users viewing printout page.*	*Number of users who viewed each of the linked menu components. Component most frequently viewed first. Doctors Questions: Dropout by page. Number of users moving back and forwards through pages. Split of users by symptom. Number of users who saw the advice pages, and type of advice given. Treatment Options & Common Questions: Number of pages viewed. Time spent in this component. Number of users who viewed each of the treatment options. Split of users by symptom.*<br>*Number of users viewing each video. Time spend on the video.*

Revisiting components/features (stage 1; 1.1, 1.2 & 2.2).	*Number of users receiving tailored feedback for low or no improvement in behavior who revisit goal setting component. Number of users returning to the start of the tunneled component from the menu page.*	*Number of users re-visiting each component and frequency of revisits. Number of re-visits during illness. Number of users revisiting components with the same/different symptoms. Number of users who login when ill more than once.*
Type of content/BCTs used (excluding administration pages) (stage 1; 1.2 & 2.2).	*User dropout by page (including consent and baseline measures pages).*	*See features/menu components*
Completing ongoing measures (stage 1; 2.1 & 2.2).	*Number of users entering their email address to take part in the survey. Number of questions completed in survey. Number of users completing each question.*	*Number of users completing monthly measures.*
External device usage (stage 1; 2.3).	*N/a*	*N/a*
**2. Relationships between usage and participant characteristics. Are user's demographic, physical or psychosocial characteristics at baseline related to intervention usage?**		
Are any characteristics at baseline related to usage?	*Is age, gender or education related to usage? Is type of household related to usage? Are perceived likelihood and/or severity for user or a member of their household related to usage* [1]*?*	*Is having a co-morbid illness related to usage? Is age associated with different patterns of usage? Is level of anxiety at baseline associated with different patterns of usage?*
Are any contextual factors related to usage (stage 1; 3)?	*Does how users hear about the website relate to usage?*	N/a
Do high/low users differ by other usage factors?	*Is there a relationship between intervention usage and survey usage?*	*Is logging in when ill related to content previously viewed? Do users who self-report GP visits use the intervention differently to users who visited their GP but didn't report it?*
**3. Relationships between usage, behavioral determinants, and target behaviors. Which usage variables are associated with follow-up measures for target behaviour and behavioral determinants? Which usage variables help explain changes in behaviour across the intervention?**		
Are baseline measures for behavioral determinants/target behavior related to usage? E.g. Is the number of days the intervention is used for related to a behavioral determinant? Do users with low target behavior spend less time on the intervention?	*Is handwashing level at baseline related to usage? Is perceived necessity or efficacy of handwashing related to usage?*	*Is the number of GP visits during year prior to trial related to usage? Are measures for health locus of control* [2]*, Krantz health opinion survey* [3]*, or TPB* [4] *attitudes/norms/intentions at baseline related to usage? Are measures for the IPQ-R* [5]*, TPB* [4] *beliefs/intentions at first login during illness related to usage during the period of illness (e.g. viewing content, completing 48hr follow-up).*
Which usage variables are related to behavioral determinants/target behaviors and at follow-up? E.g. Do users who view a group of pages	*Which usage variables are related to handwashing in the survey? Which usage variables are related to perceived necessity, and efficacy?*	*Is usage related to GP appointments? Is logging in when ill related to GP appointments? Is usage associated with the amount of times a user is ill*

containing a specific BCT score higher/lower for the associated behavioral determinant? Is completing/not completing a particular component associated with target behavior at follow-up? Is the time spent on a session related to target behavior?		*during the trial? Are measures for health locus of control* [2]*, Krantz health opinion survey* [3]*, or TPB* [4] *attitudes/norms/intentions at follow-up related to usage? Are measures for the IPQ-R* [5]*, TPB* [4] *beliefs/intentions at 48hr related to viewing content during illness?*
Is usage associated with measures for acceptability/satisfaction at follow-up? E.g. Are high levels of satisfaction associated with accessing more pages? Do users with low satisfaction spend less time using external devices?	*Which usage variables are associated with scores for user satisfaction, and acceptability e-scale measures* [6]*?*	*Are scores for patient enablement* [7]*, website satisfaction, or problematic experiences of therapy scale* [8] *related to usage?*
Do users who report positive changes in behavioral determinants/target behavior from baseline to follow-up use the intervention differently to those who do not?	*Do users who report increases in handwashing use the website differently to those who don't report increases? Do users who report increases in perceived necessity and efficacy use the intervention differently?*	*Did users with positive changes for measures for health locus of control* [2]*, Krantz health opinion survey* [3]*, or TPB* [4] *attitudes/norms/intentions use the intervention differently? Did users who reported increased positive beliefs at 48hr illness follow-up view different content during illness?*
Are relationships between usage and outcome measures moderated by demographic, psychosocial or health factors?	*Does perceived level of risk* [1] *moderate the relationship between usage and increased handwashing?*	*Does anxiety moderate relationships between usage and GP visits? Does age moderate the relationship between usage and GP visits?*
What level of usage is necessary for 'effective engagement'?	*Do any of the usage variables have a point of sufficient engagement where maximum behaviour change has occurred?*	*Did users need to see a specific amount or component of content, at a certain time in order to change their behaviour?*

1. de Zwart O, Veldhuijzen IK, Elam G, Aro AR, Abraham T, Bishop GD, Voeten HA, Richardus JH, Brug J. Perceived threat, risk perception, and efficacy beliefs related to SARS and other (emerging) infectious diseases: results of an international survey. *Int J Behav Med*. 2009;16(1):30-40. PMID: 19125335

2. Wallston KA. The validity of the multidimensional health locus of control scales. *J Health Psychol*. 2005;10(5):623-631. PMID: 16033784

3. Krantz DS, Baum A, Wideman MV. Assessment of Preferences for Self-Treatment and Information in Health Care. *J Pers Soc Psychol*. 1980;39(5):977-990. PMID: 7441487

4. Ajzen I. The theory of planned behavior. *Organ Behav Hum Decis Process*. 1991;50(2):179-211. doi:10.1016/0749-5978(91)90020-T.

5. Moss-Morris R, Weinman J, Petrie K, Horne R, Cameron L, Buick D. The Revised Illness Perception Questionnaire (IPQ-R). *Psychol Health*. 2002;17(1):1-16. doi:10.1080/08870440290001494.

6. Tariman JD, Berry DL, Halpenny B, Wolpin S, Schepp K. Validation and testing of the Acceptability E-scale for web-based patient-reported outcomes in cancer care. *Appl Nurs Res*. 2011;24(1):53-58. PMID: 20974066

7. Howie JG, Heaney DJ, Maxwell M, Walker JJ. A comparison of a Patient Enablement Instrument (PEI) against two established satisfaction scales as an outcome measure of primary care consultations. *Fam Pract*. 1998;15(2):165-171. PMID: 9613486

8. Kirby S, Donovan-Hall M, Yardley L. Measuring barriers to adherence: validation of the problematic experiences of therapy scale. *Disabil Rehabil*. 2014;36(22):1924-1929. PMID: 24410171
