# Supplementary material for: A Framework for Analyzing and Measuring Usage and Engagement Data (AMUsED) in Digital Interventions: Viewpoint
Source: J Med Internet Res. 2019 Feb 15;21(2):e10966. doi: 10.2196/10966 (PMC6396072; doi:10.2196/10966)
Supplement: Multimedia Appendix 6 [file jmir_v21i2e10966_app6.pdf]

Stage 3 checklist for the AMUsED Framework: PRIMIT Study and Internet Dr

Preparation for analysis		
Generic questions	Intervention: *Germ Defence*	Intervention: *Internet Dr*
**1. Resources**		
What is the timeframe for completing the analyses?	*By end of 2019.*	*Oct 2018.*
What resources are needed? E.g. additional research time, expertise	*At least 200 users.*<br>*Support with analyses.*	*Support with analyses, confounding variables (e.g. co morbid illnesses), and moderators. Support with visually exploring and extracting data.*
Is a plan of analysis already available? How does the analysis plan developed using the framework compare to that plan? Are changes or updates needed?	*No other plan available.*	*Primary analyses have already been carried out (see stage 1, 3.2), no further plan is available.*
Is ethical clearance in place to carry out usage analyses?	*Yes.*	*Yes.*
**2. Selecting types of analysis and analytical software**		
Will the usage data be triangulated with qualitative data?	*No.*	*No.*
What analytical tools are available?	*SPSS & LifeGuide Visualisation Tool (LVT).*	*SPSS and LifeGuide Visualisation Tool (LVT).*
Is there sufficient statistical power to answer the planned research questions?	*No, analyses will be exploratory.*	*Analyses using whole intervention group are sufficiently powered, sub-group analyses will not be.*
Can the selected measures of usage be analyzed using the available tools? Is bespoke software necessary (e.g. visualisation techniques)?	*LVT will be necessary for looking at page flow.*	*Sequence of use of components and movement through Doctors Questions will need to be analyzed using LVT.*
**3. Data preparation**		
When is the data available?	*From spring 2019.*	*Now.*
Is the data raw or has it been used/cleaned previously?	*Raw log-data.*	*Raw log-data.*
How many datasheets are there? Will these need to be amalgamated?	*8 datasheets: 4 log-data sheets for both intervention and survey. Survey data will be matched to intervention data by assigned ID. LVT: Session details will have recorded all necessary*	*5 datasheets: 4 log-data sheets, GP notes. LVT: Session details must contain important self-report measures from user data. Copy primary outcome measures, previous behaviour and co morbid*

	follow-up measures. SPSS: Page durations will need to be extracted from page durations sheet and copied to session data.	*illnesses from GP notes. SPSS: all data for analysis must be available on one data sheet. Relevant measures from GP notes will be copied to session details, along with usage variables from user data and page durations.*
Is the data structured to work with the tools available? What formats are the datasheets in (e.g. excel, .csv) and will they need converting for analysis?	*Datasheets are compatible with SPSS and LVT. Excel sheets will need converting to .csv for LVT, and uploaded to SPSS.*	*Datasheets are compatible with SPSS and LVT. Log data (session details, page flow, page durations, user data) is excel worksheet. These need to be converting to .csv for LVT. GP notes are SPSS.*
What preparation does the data need (e.g. cleaning, anonymizing)?	*Data needs cleaning.*	*Data needs cleaning.*
Are all variables readily available or will they need extracting/transforming/recoding?	*Identified variables will be available. However, totals for time and numbers of pages viewed will need extracting.*	*User data datasheet has a row of data per login so that a user who has logged in 7 times will have 7 rows of data. However, session details datasheet has only 1 row per user. Data must be transformed into 1 row in order to copy across. Totals for time spent and pages viewed will need extracting.*
Is the data in the right format to answer the research questions? Will it need adapting (e.g. continuous variables changed to categorical)?	*Handwashing, necessity, efficacy and perceived risk are continuous scales so will need changing to categorical for high/low group analysis on LVT.*	*Measures of behavioral determinants are measures as continuous variables. For comparisons of usage by high/low groups these will need to be changed to categorical.*
